# Effects of Mother’s Illness and Breastfeeding on Risk of Ebola Virus Disease in a Cohort of Very Young Children

**DOI:** 10.1371/journal.pntd.0004622

**Published:** 2016-04-08

**Authors:** Hilary Bower, Sembia Johnson, Mohamed S. Bangura, Alie Joshua Kamara, Osman Kamara, Saidu H. Mansaray, Daniel Sesay, Cecilia Turay, Francesco Checchi, Judith R. Glynn

**Affiliations:** 1 Department of Infectious Disease Epidemiology, London School of Hygiene & Tropical Medicine, London, United Kingdom; 2 Save the Children, Freetown, Sierra Leone; 3 Save the Children, London, United Kingdom; Common Heritage Foundation, NIGERIA

## Abstract

**Background:**

Young children who contract Ebola Virus Disease (EVD) have a high case fatality rate, but their sources of infection and the role of breastfeeding are unclear.

**Methods/Principal Findings:**

Household members of EVD survivors from the Kerry Town Ebola Treatment Centre in Sierra Leone were interviewed four to 10 months after discharge to establish exposure levels for all members of the household, whether or not they became ill, and including those who died. We analysed a cohort of children under three years to examine associations between maternal illness, survival and breastfeeding, and the child’s outcome. Of 77 children aged zero to two years in the households we surveyed, 43% contracted EVD. 64 children and mothers could be linked: 25/40 (63%) of those whose mother had EVD developed EVD, compared to 2/24 (8%) whose mother did not have EVD, relative risk adjusted for age, sex and other exposures (aRR) 7·6, 95%CI 2·0–29·1. Among those with mothers with EVD, the risk of EVD in the child was higher if the mother died (aRR 1·5, 0·99–2·4), but there was no increased risk associated with breast-feeding (aRR 0·75, 0·46–1·2). Excluding those breastfed by infected mothers, half (11/22) of the children with direct contact with EVD cases with wet symptoms (diarrhoea, vomiting or haemorrhage) remained well.

**Conclusion/Significance:**

This is the largest study of mother-child pairs with EVD to date, and the first attempt at assessing excess risk from breastfeeding. For young children the key exposure associated with contracting EVD was mother’s illness with EVD, with a higher risk if the mother died. Breast feeding did not confer any additional risk in this study but high risk from proximity to a sick mother supports WHO recommendations for separation. This study also found that many children did not become ill despite high exposures.

## Author Summary

Our study is the first to quantify sources of infection and describe risk of transmission of Ebola to young children. We found that the risk of a child under three developing Ebola disease was low unless their mother had EVD, and that the risk was particularly high if their mother died of EVD. But we found no additional risk from breastfeeding. WHO recommends separating asymptomatic breast-fed infants from their mothers if they develop Ebola, and using formula feeding. We support the need for separation because of the high risk related to proximity, but more research is needed to more fully understand this, particularly given the importance of breast-feeding in preventing other childhood illnesses. We also found young children in Ebola-affected households whose mothers were not ill had a surprisingly low risk of developing EVD which was not all explained by low exposure to the virus. Many children stayed well despite having direct contact with EVD patients with diarrhoea, vomiting or bleeding who are considered the most infectious. We hope these findings will provide impetus for more detailed studies into age-related response to the Ebola virus.

## Introduction

Young children experience a high case fatality rate from Ebola, but the incidence of Ebola Virus Disease (EVD) in children appears to be lower than in adults.[1–4] Young children may have limited exposure outside the home, but within the household maintaining hygiene in young children is difficult, although efforts may be made to keep children away from those who are sick. For very young children who need to be fed and held, contact with sick caregivers may be unavoidable.

Breastfeeding is a possible additional source of infection for young children: Ebola has been found in breast milk, but the risk to breastfed babies and the contribution of breastfeeding to transmission is poorly understood.[5,6] An investigation of household contacts following the Ebola outbreak in Gulu, Uganda in 2000 included five infants whose mother had EVD: three of four infants who were breastfed developed EVD.[7] The other infant was reported to have been separated from his mother early in the course of her illness and remained well; it is not clear if he was breastfed. Two recent systematic reviews of transmission of Ebola did not did not mention risks associated with breastfeeding.[8,9]

As part of a study of transmission patterns in Sierra Leone we collected data on exposure patterns and outcomes of all individuals present in the households of EVD survivors. In this analysis we sought to identify likely sources of infection and characterise risk of transmission to young children, including those breastfed by mothers with EVD.

## Methods

In July-September 2015, interviews were sought with the household members of all individuals who were discharged from the Ebola Treatment Centre in Kerry Town, Sierra Leone (“Ebola survivors”) from November 2014 to March 2015. Contact was made through members of the survivor support team who were involved in their reintegration into the community. An initial approach was made to explain the study. If the household head agreed, an interview was arranged at a community centre or other meeting place and all who were in the household at the time that members of the households had Ebola were encouraged to attend.

At the interview, individual informed written consent to participate in the study was sought from all adults, and from parents or guardians for children (< 18 years), with assent from children of 12 years or older. An inventory was drawn up of all household members who had been present in the household at the time that one or more household members were ill with EVD, including any who had died or were not present at the interview. For each member we asked whether they had had Ebola. We asked relatives whether any deceased had died of Ebola.

Household members were asked to describe what happened when Ebola came to their household, including who became ill first, whether those with Ebola had any diarrhoea, vomiting or bleeding while they were at home, and who looked after them. They were encouraged to tell the narrative in their own words, with probing questions to clarify who had been exposed and how. For each household member (including those who had died, but excluding any absent members or those who refused consent) we sought to establish the highest-risk exposure. Reported exposures were ranked a priori from highest to lowest as: contact with the body of someone who died of Ebola; direct contact with body fluids of someone with Ebola, including breastfeeding, or other direct contact with “wet” cases (i.e. those with diarrhoea, vomiting or bleeding); direct contact with “dry” cases (i.e. those without diarrhoea, vomiting or bleeding); indirect contact with a wet case (e.g. washing their clothes); indirect contact with a dry case; minimal contact (e.g. shared utensils); and no known contact. For each mother-baby pair who both had EVD we attempted to ascertain from the narratives who was affected first.

All survivors from the Kerry Town Ebola Treatment Centre had EVD confirmed by PCR. We did not have laboratory data for those from other treatment centres or for those who died, so have relied on the families’ reports. For individuals who were not reported as having had Ebola we asked about symptoms at the time that Ebola was in the household. For the analysis they were classified as not having had Ebola if they were asymptomatic or had symptoms that did not fulfil the Sierra Leone Ministry of Health and Sanitation case definition for “probable” Ebola,[10] or had had a negative test; and as having had Ebola if they were symptomatic and fulfilled the case definition for probable Ebola and were not tested. The case definition was contact with a case plus fever or miscarriage or unexplained bleeding; or contact plus three or more symptoms (of fatigue, headache, loss of appetite, nausea or vomiting, abdominal pain, diarrhoea, muscle or joint pain, sore throat or pain on swallowing, hiccups).

In this analysis we concentrate on risks to children aged less than three years at the time Ebola reached their household in order to include all those who were breast fed, and examine attack rates, case fatality rates and the role of breast feeding. Proportions were compared using Χ^2^ or Fisher’s exact test. Analyses used multivariable logistic regression. Because the outcome is very common we have presented the results as risk ratios (RR) using marginal standardization to estimate RRs, and the delta method to estimate 95% confidence intervals (95%CI).[11–13] We repeated the analysis calculating risk ratios using Poisson regression with robust error variance.[14] Crowding (number of people per room) and sanitation (access to water, soap and latrine) were considered as possible confounders, in addition to age, sex and the exposure variables. The effects of clustering by household were explored using generalised estimation equations in logistic regression: the results were very similar to analyses ignoring clustering so clustering is not included in the models. Analyses used STATA 14.

### Ethics Statement

The study was approved by the Sierra Leone Ethics and Scientific Review Committee and the Ethics Committee of the London School of Hygiene & Tropical Medicine. At the interview, individual written informed consent to participate in the study was sought from all adults, and from parents or guardians for children (< 18 years), with assent from children of 12 years or older.

## Results

One hundred and fifty one survivors were discharged from Kerry Town Ebola Treatment Centre from November 2014 through March 2015, of whom 138 were still living in the Western Area of Sierra Leone when sought for interview in July-September 2015. Twelve were uncontactable and a further two were known to have bad relationships with their households so were not approached. We contacted and interviewed 123 Kerry Town survivors, living in 94 households. Only one contacted survivor refused to be interviewed, and only two of 526 household members refused to participate. A further 37 members were not available to attend the interview. Some households also included survivors who had been treated in other facilities.

The households contained 77 children aged less than three years: 43% (33/77) got EVD, including four who fitted the case definition but were not diagnosed at the time. The risk of EVD was 54% (13/24) in those under one year; 40% (12/30) in those aged one year and 35% (8/23) in those aged two years (p-value for trend = 0.2). The risk was slightly higher in males than in females: 51·4% (18/35) vs 35·7% (15/42), p = 0.2. Three of the children were primary or co-primary cases in their household. Overall, 24 children under three years died of EVD, giving a case fatality rate of 73%: 85% (11/13), 75% (9/12) and 50% (4/8) at ages under-one, one, and two respectively (p-value for trend = 0·1).

Among the 77 children were 13 whose mothers were not present (including two mothers who had died in other households), or were not clearly identified: six (46%) of these children developed EVD and five died compared to 27 cases (42%) and 19 deaths among the 64 children who could be linked to their mothers.

Details of the mother-child pairs for whom the outcome of both mother and child are known are shown in Table 1 for the 40 whose mothers had EVD, in Table 2 for the 24 whose mothers had no symptoms, and in summary for all 64 in Table 3. The highest level of exposure is shown, in terms of direct or indirect exposure to those with EVD in the home or outside. None of the children had direct contact with dead bodies. Breastfeeding was taken as the highest exposure if the mother had EVD unless the child developed symptoms before or at the same time as the mother.

**Table 1:** Details of exposure and outcomes in mother-child pairs in which the mother had Ebola.

Child’s age in years	Mother’s outcome	Child's exposure	Other Ebola cases	No. of people in household	Child's outcome	Timing
**Breast fed**						
<1	Survivor	breastfed	4	10	Well	mother first
<1	Survivor	breastfed	6	14	Survivor	mother first
<1	Survivor	breastfed	0	12	Survivor	unclear
<1	Survivor	breastfed	15	26	Death EVD	mother first
<1	Survivor	breastfed	2	6	Death EVD	mother first
<1	Survivor	breastfed	9	15	Death EVD	mother first
<1	Death EVD	breastfed	4	10	Death EVD^1^	mother first
<1	Death EVD	breastfed	11	17	Death EVD	mother first
<1	Death EVD	breastfed	18	26	Death EVD	unclear
<1	Death EVD	breastfed	10	13	Death EVD	mother first
1	Survivor	breastfed	3	15	Well	mother first
1	Survivor	breastfed	0	3	Well	mother first
1	Survivor	breastfed	1	9	Well	mother first
1	Survivor	breastfed	9	13	Death EVD	mother first
1	Death EVD	breastfed	3	11	Well	mother first
1	Death EVD	breastfed	13	27	Survivor	mother first
**Breast fed, child ill before or same time as mother**						
1	Survivor	indirect contact wet case	1	9	Death EVD	child first
2	Survivor	direct contact wet case	4	7	Death EVD	same time
**Not breast fed**						
<1	Death EVD	direct contact wet case	12	16	Death EVD	unclear
1	SymCD/NoTest^2^	direct contact body fluids	3	11	Well	unclear
1	Survivor	direct contact body fluids	4	13	Death EVD	unclear
1	Survivor	direct contact wet case	9	15	Death EVD	mother first
1	Survivor	direct contact wet case	4	10	Well	mother first
1	Survivor	direct contact wet case	4	9	SymCD/NoTest^3^	mother first
1	Survivor	direct contact dry case	15	26	Death EVD	unclear
1	Survivor	direct contact dry case	4	12	Death EVD	unclear
1	Death EVD	direct contact dry case	15	19	Headache/ cough only	unclear
1	Death EVD	direct contact dry case	15	26	Death EVD	unclear
2	SymCD/NoTest^4^	direct contact dry case	5	15	Well	unclear
2	Survivor	direct contact wet case	3	11	Well	unclear
2	Survivor	direct contact wet case	19	26	Headache only	unclear
2	Survivor	direct contact wet case	4	7	Survivor	mother first
2	Survivor	direct contact dry case	9	14	Well	unclear
2	Survivor	direct contact dry case	1	5	SymCD/NegTest^2^	unclear
2	Survivor	indirect contact dry case	16	26	Well^5^	unclear
2	Death EVD	direct contact body fluids	5	8	SymCD/NoTest^6^	mother first
2	Death EVD	direct contact body fluids	5	8	SymCD/NoTest^6^	mother first
2	Death EVD	direct contact wet case	3	6	Death EVD	mother first
2	Death EVD	minimal contact	4	19	Well^5^	unclear
2	Death EVD	minimal contact	2	6	SymCD/NoTest^2^	mother first

Death EVD = death from Ebola; Survivor = Survived Ebola; SymCD/NoTest = fulfilled case definition for Ebola, not tested; SymCD/NegTest = fulfilled case definition for Ebola but tested negative

^1^ Possibly infected in utero or perinatally.

^2^ Fever only

^3^ Multiple symptoms, not tested because of nurses’ strike

^4^ Fever and headache

^5^ Moved out of household after first case

^6^ Multiple symptoms

**Table 2:** Details of exposures and outcomes in mother-child pairs in which the mother did not have Ebola.

Child's age (years)	Child's exposure	Other Ebola cases in household	No. of people in household	Child's outcome
<1	direct contact wet case	19	26	Death EVD
<1	direct contact wet case	14	15	Death EVD
<1	direct contact wet case	6	12	Well
<1	direct contact wet case	1	13	Well
<1	direct contact dry case	6	18	Well
<1	direct contact dry case	5	13	Well
<1	direct contact dry case	2	14	Well
<1	direct contact dry case	1	9	Well
<1	minimal contact	6	18	Well
<1	minimal contact	3	11	Well
<1	minimal contact	2	8	Well
1	direct contact wet case	6	12	Well
1	direct contact wet case	1	13	Well
1	direct contact dry case	2	11	Well
1	direct contact dry case	1	12	Well
1	direct contact dry case	1	3	Well
1	direct contact dry case	1	4	Headache only
1	minimal contact	2	7	Well
1	minimal contact	6	8	Well^1^
2	direct contact wet case	1	6	Well
2	direct contact wet case	1	13	Well
2	direct contact wet case	1	13	Well
2	direct contact dry case	2	11	Well
2	minimal contact	3	11	Well

Death EVD = death from Ebola

^1^ Moved out of household after first case

**Table 3:** Associations with Ebola in children under three years.

	Child's outcome					
	Ebola /Total	%	RR (95% CI)	RR adjusted age and sex	Full model	P (*full model*)
**All mother-baby pairs**						
Mother had Ebola						
No	2/24	8.3	1	1	1	<0.001
Yes	25/40	62.5	7.5 (1.9–28.9)	9.4 (2.6–34.0)	7.6 (2.0–29.1)^1^	
Child’s age						
< 1yr	12/22	54.6	1	1	1	0.03
1 yr	9/24	37.5	0.69 (0.36–1.3)	0.67 (0.35–1.3)	0.54 (0.33–0.88)^1^	
2 yrs	6/18	33.3	0.61 (0.29–1.3)	0.59 (0.28–1.3)	0.51 (0.27–0.96)^1^	
Child’s sex						
Female	11/31	35.5	1	1	1	0.2
Male	16/33	48.5	1.4 (0.76–2.5)	1.4 (0.80–2.6)	1.4(0.87–2.2)^1^	
Child exposure level						
breastfeeding	11/16	68.8				
direct wet	11/22	50.0				
direct dry	3/16	18.8	0.68 (0.50–0.94)^2^	0.70 (0.51–0.95)	0.93 (0.76–1.1)^1^	0.5
indirect wet	1/1	100				
indirect dry	0/1	0.0				
minimal	1/8	12.5				
**Among those with mothers with Ebola**^3^						
**All**						
Mother survived	12/24	50.0	1	1	1	
Mother died	11/14	78.6	1. 6 (0.97–2.6)	1.5 (0.98–2.3)	1.5 (0.99–2.4)^4^	0.06
**Under 2s**						
Not breastfed	7/10	70.0	1	1	1	
Breastfed	11/16	68.8	0.98 (0.58–1.7)	0.76 (0.46–1.2)	0.75 (0.46–1.2)^5^	0.3

^1^ Model included age, sex, mother’s Ebola, and exposure level

^2^ Modelled as a linear term across categories

^3^ Excluding two in which the child was ill first/at the same time

^4^ Adjusted for age, sex, and exposure level

^5^ Adjusted for age, sex, and mother's death

EVD in the children was much more likely among those whose mother had EVD (25/40, 63%) than among those whose mother did not get EVD (2/24, 8%, risk ratio (RR) 7·5, 95% confidence interval (CI) 1.9–28.9, p<0.0001, Table 3). The RR remained high after adjusting for age and sex of the child (RR 9·4, 95% CI 2·6–34·0), and after additionally adjusting for maximum exposure level (RR 7·6, 95%CI 2·0–29·1). Household crowding and sanitation were not associated with EVD in the child, and adjusting for them made little difference to the results. After adjusting for mother’s EVD status and exposure levels, the risk of EVD in the child decreased with age (Table 3). After adjusting for mother’s EVD, age, and sex, there was no effect of exposure level.

Among those whose mother had EVD, excluding the two pairs in which the children were ill first, the risk of EVD in the child was higher if the mother died (79% vs 50%, Table 3), giving a relative risk of 1·6 (95% CI 0·97–2·6). This association was similar after adjusting for the child’s age and sex and additionally for exposure level. Of the 13 children who did not get EVD whose mother survived, five had contact with the mother when she was a wet case and five only when she was a dry case (unknown for three).

As the only child over two years who was breastfed got ill at the same time as the mother and was therefore excluded, the analysis of breastfeeding was restricted to the under two’s. We also excluded the child who became ill first (Table 1), leaving 26 children. The proportion of children with EVD was very similar in those who were or were not breast fed (69% vs 70%, Table 3), RR 0·98, 0·58–1·7. There was no evidence of increased risk from breastfeeding after adjusting for age and sex (RR 0·76, 0·46–1·2) or for whether the mother died (Table 3).

The analyses were re-run excluding the six mother-child pairs for which either the mother or the child was classified as having EVD on the basis of symptoms (Table 1). The associations with having a mother with EVD (fully adjusted RR 6.5, 1·6–26·0) and with breastfeeding (fully adjusted RR 0·74 (0·47–1·2) were similar to the main analysis, but the effect of having a mother who died of Ebola was lost (fully adjusted RR 1·3, 0·76–2·1). The analyses were also rerun using Poisson regression. The results were similar to the main analysis.

Among the children under three years whose mother did not get EVD, only two children got EVD. Both were aged under one year, from households with many EVD cases (Table 2), and both were reported to have had close contact with wet cases in the household. Seven other children whose mother did not have EVD and 4 whose mother had EVD but were not breastfed, had direct contact with wet cases and did not get ill. Overall, excluding children breastfed by mothers with EVD, half (11/22) of the children who had direct contact with wet cases or fluids remained well. These contacts included sharing beds with and embracing close relatives who suffered from vomiting and/or diarrhoea.

## Discussion

Among the very young children in this study the risk of EVD depended largely on whether their mother developed EVD, with an additional risk for those whose mothers died of Ebola. The high risk in those with sick mothers is expected, and the higher risk in those with mothers who died may reflect higher viral loads and/or viral shedding in these mothers. The low risk in children in Ebola-affected households when the mother was not ill is surprising, and cannot all be explained by low exposure in the children. Overall, nearly two thirds of under-three year olds had direct contact with wet cases in the household or their body fluids. While the risk of disease decreased with decreasing exposure, half of the young children with direct exposure to wet cases remained well.

Only three children were deliberately sent out of the household to reduce exposure, and for all three there was some exposure before they left. The opportunities for households to protect children from exposure are limited, particularly as more and more cases arise, and young children share beds with sick relatives. While a ‘no touch policy’ may be understood by older children, it is impossible to explain to an infant.

Among children whose mothers had EVD, being breastfed did not appear to increase the risk. Numbers were small and risks were already high in this group so there was limited power to detect an association. Current WHO guidelines recommend that asymptomatic breastfed infants of Ebola-infected mothers should be separated from their mothers and replacement fed.[15] Although we found no excess risk from breastfeeding, further studies, ideally with larger, pooled datasets, are needed to assess this further before suggesting any changes to the recommendation. The high risk from proximity to a sick mother supports the need for separation.

The children in this study all came from households with at least one survivor. This may mean small households and households with fewer cases are underrepresented, as there would be a lower chance for small households to include a survivor, and households in which all cases of EVD died are missed. This might underestimate the case fatality rate and overestimate attack rates, but should not bias the relative risks by age and exposure.

This study shows the remarkable resilience of some young children despite apparent exposure to Ebola. This could be dose-related—we do not know the actual viral exposure through contact or breastfeeding—but in other contexts some people seem to be infected from minimal exposures. Relative resistance to Ebola could be influenced by genetic factors,[16] though the correlation between infections in mothers and children is more likely to reflect exposure patterns than shared genes. It is possible that there is some protection through maternal antibody from breastfeeding (perhaps more in mothers who survive) that counteracts any increased risk from transmission via breastmilk.

This is much the largest study of mother-child pairs with EVD to date, and the first attempt to assess any excess risk from breastfeeding. By visiting households after transmission had ceased and talking to all members we were able to determine exposure in much more detail than is possible in an acute epidemic situation. And because we included all children in these households, including those who were not sick, we have been able to calculate age and exposure-specific attack rates. In these households the risk to young children was largely dependent on whether their mother had EVD, regardless of whether they were breastfed.

## Supporting Information

S1 File STROBE checklist. Completed STROBE checklist.
(DOCX)
