# Supplementary material for: Effects of Mother’s Illness and Breastfeeding on Risk of Ebola Virus Disease in a Cohort of Very Young Children
Source: PLoS Negl Trop Dis. 2016 Apr 8;10(4):e0004622. doi: 10.1371/journal.pntd.0004622 (PMC4825998; doi:10.1371/journal.pntd.0004622)
Supplement: S1 File — Completed STROBE checklist. (DOCX) [file pntd.0004622.s001.docx]

STROBE Statement—checklist of items that should be included in reports of observational studies

|  | Item No. | Recommendation | Page  No. | Relevant text from manuscript |
| --- | --- | --- | --- | --- |
| **Title and abstract** | 1 | (*a*) Indicate the study’s design with a commonly used term in the title or the abstract | p1 | **Lines 1-2:** Effects of mother’s illness and breastfeeding on risk of Ebola Virus Disease in a cohort of very young children |
|  |  | (*b*) Provide in the abstract an informative and balanced summary of what was done and what was found | p2 | **Lines 30-34**: Household members of EVD survivors from the Kerry Town Ebola Treatment Centre in Sierra Leone were interviewed four to 10 months after discharge to establish exposure levels for all members of the household, whether or not they became ill, and including those who died. We analysed a cohort of children under three years to examine associations between maternal illness, survival and breastfeeding, and the child’s outcome.  **Lines 35-42:** Of 77 children aged 0-2 years in the households we surveyed, 43% contracted EVD. 64 children and mothers could be linked: 25/40 (63%) of those whose mother had EVD developed EVD, compared to 2/24 (8%) whose mother did not have EVD, relative risk adjusted for age, sex and other exposures (aRR) 7·6, 95%CI 2·0-29·1. Among those with mothers with EVD, the risk of EVD in the child was higher if the mother died (aRR 1·5, 0·99-2·4), but there was no increased risk associated with breast-feeding (aRR 0·75, 0·46-1·2). Excluding those breastfed by infected mothers, half (11/22) of the children with direct contact with EVD cases with wet symptoms (diarrhoea, vomiting or haemorrhage) remained well. |
| Introduction | | | |  |
| Background/rationale | 2 | Explain the scientific background and rationale for the investigation being reported | P4, | **Line 65-72**: Young children experience a high case fatality rate from Ebola, but the incidence of Ebola Virus Disease (EVD) in children appears to be lower than in adults.[^1-4^](#_ENREF_1) Young children may have limited exposure outside the home, but within the household maintaining hygiene in young children is difficult, although efforts may be made to keep children away from those who are sick. For very young children who need to be fed and held, contact with sick caregivers may be unavoidable.  Breastfeeding is a possible additional source of infection for young children: Ebola has been found in breast milk, but the risk to breastfed babies and the contribution of breastfeeding to transmission is poorly understood.[^5^](#_ENREF_5)^,^[^6^](#_ENREF_6) |
| Objectives | 3 | State specific objectives, including any prespecified hypotheses | P4, | **Line 79-81:** we sought to identify likely sources of infection and characterise risk of transmission to young children, including those breastfed by mothers with EVD.  Line 127-8:… examine attack rates, case fatality rates and the role of breast feeding. |
| Methods | | | |  |
| Study design | 4 | Present key elements of study design early in the paper | P4-5, | **Line 83-89**: …interviews were sought with the household members of all individuals who were discharged from the Ebola Treatment Centre in Kerry Town, Sierra Leone … who were in the household at the time that members of the households had Ebola..  **Line 90-98** At the interview, individual written consent to participate in the study was sought from all adults, and from parents or guardians for children (< 18 years), with assent from children of 12 years or older. An inventory was drawn up of all household members who had been present in the household at the time that one or more household members were ill with EVD, including any who had died or were not present at the interview….  For each member we asked whether they had had Ebola or whether they had died of Ebola.  Household members were asked to describe what happened when Ebola came to their household, including who became ill first, whether those with Ebola had any diarrhoea, vomiting or bleeding while they were at home, and who looked after them. ….  **Line 100-102**: For each household member (including those who had died, but excluding any absent members or those who refused consent) we sought to establish the highest-risk exposure. Reported exposures were ranked a priori from highest to lowest...  **Line 107-108** For each mother-baby pair who both had EVD we attempted to ascertain from the narratives who was affected first. |
| Setting | 5 | Describe the setting, locations, and relevant dates, including periods of recruitment, exposure, follow-up, and data collection | P4 | **Lines 83-85:** In july-September 2015, … interviews were sought with the household members of all individuals who were discharged from the Ebola Treatment Centre in Kerry Town, Sierra Leone…from November 2014 to March 2015  **Lines 96-98**: Household members were asked to describe what happened when Ebola came to their household, including who became ill first, whether those with Ebola had any diarrhoea, vomiting or bleeding while they were at home, and who looked after them. …. |
| Participants | 6 | (*a*) *Cohort study*—Give the eligibility criteria, and the sources and methods of selection of participants. Describe methods of follow-up | P4-5 | **Lines 83-84**: …the household members of all individuals who were discharged from the Ebola Treatment Centre in Kerry Town …from November 2014 – March 2015.  **Lines 120-122:** In this analysis we concentrate on risks to children aged less than three years at the time Ebola reached their household…  **Lines 96-99**: Household members were asked to describe what happened when Ebola came to their household, including who became ill first, whether those with Ebola had any diarrhoea, vomiting or bleeding while they were at home, and who looked after them. They were encouraged to tell the narrative in their own words,… |
|  |  | (*b*) *Cohort study*—For matched studies, give matching criteria and number of exposed and unexposed  *Case-control study*—For matched studies, give matching criteria and the number of controls per case |  | n/a  n/a |
| Variables | 7 | Clearly define all outcomes, exposures, predictors, potential confounders, and effect modifiers. Give diagnostic criteria, if applicable | P5-6 | **Lines 109-119** (outcomes): All survivors from the Kerry Town Ebola Treatment Centre had EVD confirmed by PCR. We did not have laboratory data for those from other treatment centres or for those who died, so have relied on the families’ reports. For individuals who were not reported as having had Ebola we asked about symptoms at the time that Ebola was in the household. For the analysis they were classified as not having had Ebola if they were asymptomatic or had symptoms that did not fulfil the Sierra Leone Ministry of Health and Sanitation case definition for “probable” Ebola,[^10^](#_ENREF_10) or had had a negative test; and as having had Ebola if they were symptomatic and fulfilled the case definition for probable Ebola and were not tested. The case definition was contact with a case plus fever or miscarriage or unexplained bleeding; or contact plus three or more symptoms (of fatigue, headache, loss of appetite, nausea or vomiting, abdominal pain, diarrhoea, muscle or joint pain, sore throat or pain on swallowing, hiccups).  **Lines 96-99** (exposures): Household members were asked to describe what happened when Ebola came to their household, including who became ill first, whether those with Ebola had any diarrhoea, vomiting or bleeding while they were at home, and who looked after them….  **Lines 126-130** (confounders): Crowding (number of people per room) and sanitation (access to water, soap and latrine) were considered as possible confounders, in addition to age, sex and the exposure variables. The effects of clustering by household were explored... |
| Data sources/ measurement | 8* | For each variable of interest, give sources of data and details of methods of assessment (measurement). Describe comparability of assessment methods if there is more than one group | P5 | *Data Source: as noted above; interviews*  *Exposure level:* **Lines 101-108:**  Reported exposures were ranked a priori from highest to lowest as: contact with the body of someone who died of Ebola; direct contact with body fluids of someone with Ebola, including breastfeeding, or other direct contact with “wet” cases (i.e. those with diarrhoea, vomiting or bleeding); direct contact with “dry” cases (i.e. those without diarrhoea, vomiting or bleeding); indirect contact with a wet case (e.g. washing their clothes); indirect contact with a dry case; minimal contact (e.g. shared utensils); and no known contact. For each mother-baby pair who both had EVD we attempted to ascertain from the narratives who was affected first. |
| Bias | 9 | Describe any efforts to address potential sources of bias | P5  P13  P5-6 | *Selection bias1:* **Line 88-89**: all who were in the household at the time that members of the households had Ebola were encouraged to attend.  *Selection bias2.* ***Lines 239-243:*** The children in this study all came from households with at least one survivor. This may mean small households and households with fewer cases are underrepresented, as there would be a lower chance for small households to include a survivor, and households in which all cases of EVD died are missed. This might underestimate the case fatality rate and overestimate attack rates, but should not bias the relative risks by age and exposure.  *Selection bias3 - refusals reduced:* **lines 134-141**: One hundred and fifty one survivors were discharged…of whom 138 were still living in the Western Area of Sierra Leone when sought for interview in July-September 2015. Twelve were uncontactable and a further two were known to have bad relationships with their households so were not approached. We contacted and interviewed 123 Kerry Town survivors, living in 94 households. Only one contacted survivor refused to be interviewed, and only two of 526 household members refused to participate. … 37 members were not available to attend the interview.  *Confounding*: **Lines 124-130** Crowding (number of people per room) and sanitation (access to water, soap and latrine) were considered as possible confounders, in addition to age, sex and the exposure variables. The effects of clustering by household were explored...  *Recall bias:* **lines 96-99** : Household members were asked to describe what happened when Ebola came to their household, … They were encouraged to tell the narrative in their own words, with probing questions to clarify … |
| Study size | 10 | Explain how the study size was arrived at |  | *Available data:* **lines 83-86:** interviews were sought with the household members of all individuals who were discharged from the Ebola Treatment Centre in Kerry Town, Sierra Leone (“Ebola survivors”) from November 2014 to March 2015 |

| Quantitative variables | 11 | Explain how quantitative variables were handled in the analyses. If applicable, describe which groupings were chosen and why |  | n/a |
| --- | --- | --- | --- | --- |
| Statistical methods | 12 | (*a*) Describe all statistical methods, including those used to control for confounding | P6 | **Lines 122-130:** Proportions were compared using Χ^2^ or Fisher’s exact test. Analyses used multivariable logistic regression. Because the outcome is very common we have presented the results as risk ratios (RR) using marginal standardization to estimate RRs, and the delta method to estimate 95% confidence intervals (95%CI).[^11-13^](#_ENREF_11) We repeated the analysis calculating risk ratios using Poisson regression with robust error variance.[^14^](#_ENREF_14) Crowding (number of people per room) and sanitation (access to water, soap and latrine) were considered as possible confounders, in addition to age, sex and the exposure variables. The effects of clustering by household were explored using generalised estimation equations in logistic regression: the results were very similar to analyses ignoring clustering so clustering is not included in the models. |
|  |  | (*b*) Describe any methods used to examine subgroups and interactions | P7 | **Lines 149-152:** Among the 77 children were 13 whose mothers were not present (including two mothers who had died in other households), or were not clearly identified: six (46%) of these children developed EVD and five died compared to 27 cases (42%) and 19 deaths among the 64 children who could be linked to their mothers. |
|  |  | (*c*) Explain how missing data were addressed |  | *Excluded from main analysis*: **Line 150-156:**  Among the 77 children were 13 whose mothers were not present (including two mothers who had died in other households), or were not clearly identified: six (46%) of these children developed EVD and five died compared to 27 cases (42%) and 19 deaths among the 64 children who could be linked to their mothers.  Details of the mother-child pairs for whom the outcome of both mother and child are known are shown in table 1 for the 40 whose mothers had EVD, in table 2 for the 24 whose mothers had no symptoms, and in summary for all 64 in table 3. |
|  |  | (*d*) *Cohort study*—If applicable, explain how loss to follow-up was addressed  *Case-control study*—If applicable, explain how matching of cases and controls was addressed  *Cross-sectional study*—If applicable, describe analytical methods taking account of sampling strategy |  | n/a |
|  |  | (*e*) Describe any sensitivity analyses | P11 | **Lines 205-208:** The analyses were re-run excluding the six mother-child pairs for which either the mother or the child was classified as having EVD on the basis of symptoms (table 1). The associations with having a mother with EVD (fully adjusted RR 6.5, 1·6-26·0) and with breastfeeding (fully adjusted RR 0·74 (0·47-1·2) were similar to the main analysis, but the effect of having a mother who died of Ebola was lost (fully adjusted RR 1·3, 0·76-2·1). The analyses were also rerun using Poisson regression. The results were similar to the main analysis. |
| Results | | | | |
| Participants | 13* | (a) Report numbers of individuals at each stage of study—eg numbers potentially eligible, examined for eligibility, confirmed eligible, included in the study, completing follow-up, and analysed | P6-7  P10-11 | **Lines 137-143**: One hundred and fifty one survivors were discharged from Kerry Town Ebola Treatment Centre from November 2014 through March 2015, of whom 138 were still living in the Western Area of Sierra Leone when sought for interview in July-September 2015. Twelve were uncontactable and a further two were known to have bad relationships with their households so were not approached. We contacted and interviewed 123 Kerry Town survivors, living in 94 households. Only one contacted survivor refused to be interviewed, and only two of 526 household members refused to participate. A further 37 members were not available to attend the interview.  **Line 145:** The households contained 77 children aged less than three years  **Lines 152-158:** Among the 77 children were 13 whose mothers were not present…Details of the mother-child pairs for whom the outcome of both mother and child are known are shown in table 1 for the 40 whose mothers had EVD, in table 2 for the 24 whose mothers had no symptoms, and in summary for all 64 in table 3.  **Line 197-198:** As the only child over two years who was breastfed got ill at the same time as the mother and was therefore excluded, the analysis of breastfeeding was restricted to the under two’s. |
|  |  | (b) Give reasons for non-participation at each stage | P 6-7 | **Lines 138-143:** …of whom 138 were still living in the Western Area of Sierra Leone when sought for interview in July-September 2015. Twelve were uncontactable and a further two were known to have bad relationships with their households so were not approached. ….Only one contacted survivor refused to be interviewed, and only two of 526 household members refused to participate. A further 37 members were not available to attend the interview |
|  |  | (c) Consider use of a flow diagram |  | *Not considered necessary* |
| Descriptive data | 14* | (a) Give characteristics of study participants (eg demographic, clinical, social) and information on exposures and potential confounders | P8-10 | *Please see tables 1-2-3* |
|  |  | (b) Indicate number of participants with missing data for each variable of interest | P7 | **Line 152-155***:* Among the 77 children were 13 whose mothers were not present (including two mothers who had died in other households), or were not clearly identified: |
|  |  | (c) *Cohort study*—Summarise follow-up time (eg, average and total amount) |  | *Varies by household because related to the period of time that the household had ebola* |
| Outcome data | 15* | *Cohort study*—Report numbers of outcome events or summary measures over time | P8-10 | *Please see tables 1-2-3* |
|  |  | *Case-control study—*Report numbers in each exposure category, or summary measures of exposure |  | *n/a* |
|  |  | *Cross-sectional study—*Report numbers of outcome events or summary measures |  | *n/a* |
| Main results | 16 | (*a*) Give unadjusted estimates and, if applicable, confounder-adjusted estimates and their precision (eg, 95% confidence interval). Make clear which confounders were adjusted for and why they were included | P10 | *Please see table 3*  **Line 192-201:** The RR remained high after adjusting for age and sex of the child (RR 9·4, 95% CI 2·6-34·0), and after additionally adjusting for maximum exposure level (RR 7·6, 95%CI 2·0-29·1). Household crowding and sanitation were not associated with EVD in the child, and adjusting for them made little difference to the results. After adjusting for mother’s EVD status and exposure levels, the risk of EVD in the child decreased with age (table 3). After adjusting for mother’s EVD, age, and sex, there was no effect of exposure level.  Among those whose mother had EVD, excluding the two pairs in which the children were ill first, the risk of EVD in the child was higher if the mother died (79% vs 50%, table 3), giving a relative risk of 1·6 (95% CI 0·97-2·6). This association was similar after adjusting for the child’s age and sex and additionally for exposure level.  **Lines 207-209**: The proportion of children with EVD was very similar in those who were or were not breast fed (69% vs 70%, table 3), RR 0·98, 0·58-1·7. There was no evidence of increased risk from breastfeeding after adjusting for age and sex (RR 0·76, 0·46-1·2) or for whether the mother died (table 3). |
|  |  | (*b*) Report category boundaries when continuous variables were categorized | p.8-10 | *Please see tables 1-2-3 for age category boundaries* |
|  |  | (*c*) If relevant, consider translating estimates of relative risk into absolute risk for a meaningful time period |  | n/a |

| Other analyses | 17 | Report other analyses done—eg analyses of subgroups and interactions, and sensitivity analyses | P11 | *Sensitvity analysis as described above*  **Lines 203-208:** The analyses were re-run excluding the six mother-child pairs for which either the mother or the child was classified as having EVD on the basis of symptoms (table 1). The associations with having a mother with EVD (fully adjusted RR 6.5, 1·6-26·0) and with breastfeeding (fully adjusted RR 0·74 (0·47-1·2) were similar to the main analysis, but the effect of having a mother who died of Ebola was lost (fully adjusted RR 1·3, 0·76-2·1). The analyses were also rerun using Poisson regression. The results were similar to the main analysis. |
| --- | --- | --- | --- | --- |
| Discussion | | | | |
| Key results | 18 | Summarise key results with reference to study objectives | P12 | **Lines 218-225**Among the very young children in this study the risk of EVD depended largely on whether their mother developed EVD, with an additional risk for those whose mothers died of Ebola. The high risk in those with sick mothers is expected, and the higher risk in those with mothers who died may reflect higher viral loads and/or viral shedding in these mothers. The low risk in children in Ebola-affected households when the mother was not ill is surprising, and cannot all be explained by low exposure in the children. Overall, nearly two thirds of under-three year olds had direct contact with wet cases in the household or their body fluids. While the risk of disease decreased with decreasing exposure, half of the young children with direct exposure to wet cases remained well.  **Lines 231-233 :** Among children whose mothers had EVD, being breastfed did not appear to increase the risk. |
| Limitations | 19 | Discuss limitations of the study, taking into account sources of potential bias or imprecision. Discuss both direction and magnitude of any potential bias | P13 | **Lines 231-233** : Among children whose mothers had EVD, being breastfed did not appear to increase the risk. Numbers were small and risks were already high in this group so there was limited power to detect an association.  **Line 238-242:** The children in this study all came from households with at least one survivor. This may mean small households and households with fewer cases are underrepresented, as there would be a lower chance for small households to include a survivor, and households in which all cases of EVD died are missed. This might underestimate the case fatality rate and overestimate attack rates, but should not bias the relative risks by age and exposure. |
| Interpretation | 20 | Give a cautious overall interpretation of results considering objectives, limitations, multiplicity of analyses, results from similar studies, and other relevant evidence | P13 | **Line 243-249:** This study shows the remarkable resilience of some young children despite apparent exposure to Ebola. This could be dose-related – we do not know the actual viral exposure through contact or breastfeeding – but in other contexts some people seem to be infected from minimal exposures. Relative resistance to Ebola could be influenced by genetic factors,[^16^](#_ENREF_16) though the correlation between infections in mothers and children is more likely to reflect exposure patterns than shared genes. It is possible that there is some protection through maternal antibody from breastfeeding (perhaps more in mothers who survive) that counteracts any increased risk from transmission via breastmilk.  **Lines 262-264:** In these households the risk to young children was largely dependent on whether their mother had EVD, regardless of whether they were breastfed. |
| Generalisability | 21 | Discuss the generalisability (external validity) of the study results | P13 | **Line 250-251:** This is much the largest study of mother-child pairs with EVD to date, and the first attempt to assess any excess risk from breastfeeding. |
| Other information | |  | | |
| Funding | 22 | Give the source of funding and the role of the funders for the present study and, if applicable, for the original study on which the present article is based |  | The study was funded by Save the Children internal funds and The Wellcome Trust's Enhancing Research Activity in Epidemic Situations (ERAES) programme.  SCI operated the Kerry Town Ebola Treatment Centre during the period under study, and employed the field team members. One author (FC) is employed by Save the Children UK and was involved in commissioning the study and interpreting findings. Final responsibility for the findings and paper lies with the LSHTM authoris who had no involvement with the Kerrytown ETC or any SCI activities. |

*Give information separately for cases and controls in case-control studies and, if applicable, for exposed and unexposed groups in cohort and cross-sectional studies.

**Note:** An Explanation and Elaboration article discusses each checklist item and gives methodological background and published examples of transparent reporting. The STROBE checklist is best used in conjunction with this article (freely available on the Web sites of PLoS Medicine at http://www.plosmedicine.org/, Annals of Internal Medicine at http://www.annals.org/, and Epidemiology at http://www.epidem.com/). Information on the STROBE Initiative is available at www.strobe-statement.org.
